# Notes on Preparations for the Sick

**Published:** 1907-06

**Authors:** 


### Notes on Preparations for the Sick.

For any chemical analyses mentioned in the following report we are indebted to Mr. O. C. M. Davis, B.Sc., A.I.C., of the University College Laboratory.

**The "British Pharmaceutical Codex: an Imperial Dispensatory."**—An ideal pharmacopœia should satisfy three different requirements. First, it should contain all the drugs and chemicals in general use; secondly, it should contain such combinations of these as are commonly prescribed by medical men; and thirdly, it should serve as a standard for purity of drugs and chemicals, which will be recognised by those persons responsible for the sale and preparation of these articles.

In order to in any degree comply with these three requirements it is obviously necessary that a national pharmacopœia, such, for example, as the British Pharmacopœia, shall confine itself strictly to those drugs, chemicals and formulæ which are the most universally recognised and prescribed. It necessarily leaves to other and non-official formularies a multitude of formulæ of all kinds that, while in frequent use, are not in universal use.

From this arises one of the difficulties of modern prescribing. A prescriber wishes to order, perhaps, "Spirit Soap." He has been accustomed in his hospital work to order it and get a certain preparation. If he orders it in a different part of the country he is never sure of getting the same article unless he can specify the source of the formulæ on the prescription.

There are, of course, a number of excellent formularies in common use, "Squire" and "Martindale," for example, and these to some extent meet the difficulty.

The British Pharmaceutical Society are now preparing a work, to be termed the "British Pharmaceutical Codex," which will shortly be published, which will, it is hoped, meet all requirements. It will contain, besides drugs and chemicals, formulæ for acids, baths, bougies, cerates, drops, effervescent granules, elixirs, emulsions, enemas, gargles, gauzes, insufflations, mixtures, paints, tablets, &c, &c., &c. The galenical portion of the book will therefore include more preparations than any pharmacopœia yet published, and will therefore combine the features of a hospital pharmacopœia with those of the B. P.

It is hoped that every medical man will use the book, and prescribe from it. If he then order "Elixir—(Codex)," he will ensure always getting the same preparation dispensed without the delay entailed by the pharmacist having to ring him up before dispensing the prescription.

The book can now be ordered in advance, at 10s. 6d. per copy, from the Pharmaceutical Society, or, if desired, through the pharmacist at the Royal Infirmary. As soon as published, a copy can be seen at the Dispensary of the latter institution.

---

**Alaxa.**—Burroughs, Wellcome & Co., London.—Alaxa is an aromatic liqueur of cascara sagrada, which presents, in a most pleasant and acceptable condition, the laxative properties of the bark, in combination with stomachic and carminative principles. It is of agreeable flavour, and it exerts a marked tonic effect upon the bowel; it assures a normal activity, and renders unnecessary the use of after-dinner pills or digestive aids.

Alaxa is eminently suitable for use in the treatment of the constipation of pregnancy. It regulates the action of the bowel without producing irritation or griping. Whilst purgatives may adversely affect the course of pregnancy, the tonic laxative properties of alaxa maintain the normal bowel action, and prevent interference with the gravid uterus.

The dose is one to two fluid drachms, as may be required.

---

"**Elixoid" Pine Tar Compound.**—Burroughs, Wellcome & Co., London.—A pleasantly-flavoured fluid preparation, containing pinol, tar, terpin hydrate, Virginian prune, balsam of Tolu, and ipecacuanha. This combination is very effective in the treatment of affections of the respiratory organs, as its active components are excreted by the lungs, and thus exert a slow, steady and continuous action. It is believed to allay pulmonary irritation, and is of special service in chronic bronchitis and bronchorrhœa; it also relieves coughs, and may be used in cases in which the preparations of opium are inadvisable. Half to two fluid drachms may be given three or four times a day after meals.

**Nizin.**—BURROUGHS, WELLCOME & Co., London.—Under this name Messrs. Burroughs and Wellcome have introduced the zinc salt of sulphanilic acid, which is recommended for preparing antiseptic solutions and injections.

The antiseptic value of the sulphonic acids has been recognised for many years, and zinc-sulphocarbolate (the zinc salt of phenol-para-sulphonic acid) was included in the British Pharmacopœia as far back as 1885.

Sulphanilic acid (amido-benzene-para-sulphonic acid) differs from the phenol-sulphonic acids by the substitution of the amido group ($-NH^2$) for the hydroxyl group (-OH), and this small difference may cause a very profound change in the pharmacological action.

Whether Nizin is superior to zinc-sulphocarbolate or not must be determined by clinical observations, but we imagine it will prove equally valuable as an antiseptic, and less liable to cause irritation. The Soloid (gr. 7) affords a ready means of preparing fresh solutions for urethral injections and for eye-lotions.

